# Green Jobs—A Literature Review

**DOI:** 10.3390/ijerph19137998

**Published:** 2022-06-29

**Authors:** Mihaela-Roberta Stanef-Puică, Liana Badea, George-Laurențiu Șerban-Oprescu, Anca-Teodora Șerban-Oprescu, Laurențiu-Gabriel Frâncu, Alina Crețu

**Affiliations:** 1 Department of Economics and Economic Policies, Bucharest University of Economic Studies, 6 Romana Sq., District 1, 010734 Bucharest, Romania; mihaela.stanef@economie.ase.ro; 2 Department of Economic Doctrines and Communication, Bucharest University of Economic Studies, 6 Romana Sq., District 1, 010734 Bucharest, Romania; george.serban@economie.ase.ro (G.-L.Ș.-O.); laurentiu.francu@economie.ase.ro (L.-G.F.); cretu.alina@economie.ase.ro (A.C.); 3 Department of Modern Languages and Business Communication, Bucharest University of Economic Studies, 6 Romana Sq., District 1, 010734 Bucharest, Romania; teodora.oprescu@rei.ase.ro

**Keywords:** green jobs, environment, labor market

## Abstract

In the past two decades the topic of “green jobs” has drawn particular attention, resulting in a widely diverse and relatively large number of published papers. Although a determinant for the increase of knowledge, the heterogeneity of these studies may raise the issue of a systematic documentation of the key contributions in this field. In this context, the present research focuses on analyzing the scientific literature published in the last five years on the topic of “green jobs” with the aim to identify definitions and meanings associated with the concept of “green jobs”, the connected terms, areas of research interest and the main theoretical and practical results. The results reveal that although there is no uniformity in the definition of the concept, there is still a convergence towards the initial meaning offered by the UNEP/ILO/IOE/ITUC Report of 2008. Moreover, using scientific software VOSViewer our research shows that the concept of “green jobs” is most linked with the following terms: sustainable development, the green economy, the circular economy, the welfare economy, the European Green Pact, energy, renewable energy, economic development, and employment. Furthermore, our analysis reveals that the studies focused on “green jobs” are mainly concerned with the following issues: green jobs creation, work-life balance, correlations between green business and green jobs and the role of local government in supporting green jobs.

## 1. Introduction

In recent years, one of the most debated topics among scholars, as well as policymakers, has related to climate change and the most efficient response to its detrimental consequences. According to the European Commission (2019), the global atmosphere is warming and that may cause the extinction of no less than one million out of eight million species [1]. In this context, climate change and degradation of the natural environment at the same time have been recognized as global factors for change. Thus, an important step to fight this phenomenon was taken in 2019 with adoption of the Green Deal. This is seen as a new growth strategy decoupled from the use of resources; one which will seek a stop to the production of net greenhouse gas emissions until 2050, aim at conserving the EU’s natural capital and to protect the health and well-being of its citizens from the challenges posed by detrimental impacts on the environment [1].

Given the emergence of “black swan” surprises, such as the COVID-19 pandemic and the war in Ukraine, it is becoming more than necessary to implement measures that will lead to a successful Green Deal, considering the application of the principles of solidarity, sustainable development, and well-being. In this context, it has become more evident than ever that there is an urgent need to move forward with an older initiative—the Green Jobs Initiative. “The Green Jobs Initiative” emerged as a result of a partnership between the United Nations Environment Program (UNEP), the International Trade Union Confederation (ITUC), the International Organization of Employers (IOE) and the International Labor Organization (ILO). The main goal of this partnership is the promotion of opportunities, equity, and a fair transition to sustainable economies [2]. The first report published in 2008 defines “green jobs” as any decent work that contributes to maintaining and restoring the quality of the environment, whether it is agriculture, industry, services, or administration. This outcome may be achieved by reducing energy consumption and raw materials, minimizing pollution and waste, protecting, and restoring ecosystems and enabling companies and communities to adapt to climate change [2].

Over time, creating green jobs has come to be seen as a possible solution for creating new jobs, while the green economy has come to be seen as a solution to climate change, environmental degradation and poverty growth.

Using color coding to create a more vivid picture of the labor market is not new. One can find examples in the literature referring to professions that are white, blue, pink, gold and green [3]. According to [3], green collar workers could include individuals who practice professions encompassing sustainable development principles in the upgrading of processes (for instance: jobs in the public transport sector, renewable energy sources, construction and waste management).

The studies related to “green job” concepts are usually conducted in two ways—either qualitative or quantitative. The quantitative approach mainly revolves around the idea of designing econometric models based on variables describing the number of green jobs in national economies, while the qualitative approach describes green jobs in terms of the specific skills needed to perform tasks [4].

Moreover, the definition of “green jobs” is approached in various ways in the existing literature and there are significant discrepancies when it comes to issues such as the economic importance of jobs, environmental protection, equality and social justice or sustainable development principles [3,5].

Nevertheless, one thing is certain, green jobs lead to the creation of a new management framework based on the need to connect to the eco-efficient technologies of the future and to use resources efficiently in an effort to reduce environmental pollution and climate change [3]. In addition, green jobs require decent work [2], connecting the 1st Millennium Development Goal—poverty reduction—with the 7th Millennium Development Goal—environmental protection.

In addition, the European Commission [6] defines a green job as being ”one that directly deals with information, technologies, or materials that preserves or restores environmental quality. This requires specialized skills, knowledge, training, or experience (e.g., verifying compliance with environmental legislation, monitoring resource efficiency within the company, promoting and selling green products and services)”. However, different economists formulate various definitions of green jobs. Hence, Colijn propose a slightly different definition of green jobs: ”A green job features characteristics that contribute to a socio-ecological transition in focus and activity through supporting an increase in the use of renewable energy or a reduction of the use of non-renewable energy” [7]. This definition actually proposes the use of several shades of green, depending on the involvement in the socio-ecological transition, being assigned a certain shade of green to each type of occupation depending on the extent to which work activities can be considered “green”.

There is no uniformity in the literature concerning the specific areas in which green jobs can be created. Therefore, the economic sectors prone to green job creation are differently showcased at the international level. For instance, the Bureau of Labor Statistics identifies activities related to pollution reduction and recycling, or activities specific to organic farms and non-energy activities [3]. On the other hand, the UNEP mentions broader sectors such as agriculture, production, research and development, renewable energy, services and administration and other sectors of the economy with a substantial participation in maintaining or restoring environmental quality [3].

Against this background, the present paper builds on the previous work of a significant number of researchers and primarily attempts to provide guidance on the identification of current definitions as well as an anticipation of future definitions and how green jobs are perceived in diverse sectors of the economy.

## 2. Materials and Methods

The increased interest in sustainable development and the special emphasis on environmental protection have led to the development and publication in the last decade of a significant number of studies that have focused on topics such as sustainability, education for sustainability, green economy, green innovation, circular economy, sustainable entrepreneurship and, more recently and particularly, the concept of green jobs. The variety of issues addressed as well as the large number of published studies are undoubtedly positive aspects, but at the same time can raise several issues in the unitary identification of contributions to increasing knowledge in this field. In such a context, the defining role of a literature review is to systematize existing knowledge in a particular field to facilitate its use in further studies and research. In addition, the identification, synthetization, and analysis of existing studies allow for a deeper understanding of the field and may contribute to the development of new hypotheses or more rigorous testing of existing theories [8].

In terms of sustainability in general and of the concepts traditionally associated with it, there are a number of current reviews in the literature [9,10,11,12], to which we may add a study on green human resource management [13]. However, in the case of the specific concept of green jobs, a systematic analysis of the studies published so far in this field could not be identified. In this context, the main objective of our research is to analyze the scientific literature to identify the definitions and meanings given to the term “green jobs”, the concepts associated with it, the research areas in which the concept is frequently encountered, and also the main theoretical and practical results obtained in the last 5 years in the research studies conducted on green jobs.

From a methodological perspective, in accordance with the research objective, the analysis included the following steps: (1) identifying the studies published and indexed in the Web of Science—Core Collection and Scopus databases—and selecting the works considered to be the most relevant for our analysis; (2) critical analysis of the selected articles from the perspective of their definition for the concept “green jobs”, the field of interest and the main results obtained; and (3) synthesis and interpretation of the results obtained following the analysis of selected articles from the research literature.

In the first stage, a scan of the papers published since 2008 in the Web of Science—Core Collection and Scopus databases—was performed using as a search criterion the identification of the concept “green jobs” in the title, abstract or keywords and selecting only the “article” type. The two databases were chosen because they contain the richest collection of scientific papers that have undergone a rigorous review process before being published and are the most relevant in that field. Web of Science is the oldest database [14] and it includes 10,000 journals, while Scopus is the largest database for multidisciplinary scientific literature [15]. The search period has been fixed since 2008 because in this specific year the first explicit definition of the concept “green jobs” was identified [2].

Information concerning the number of articles identified following the application of the search criteria in the two databases, by publication period, is presented in Table 1. Given that most papers can be indexed in both databases, a cumulation of numbers would not be relevant and could be misleading.

**Figure 1:**
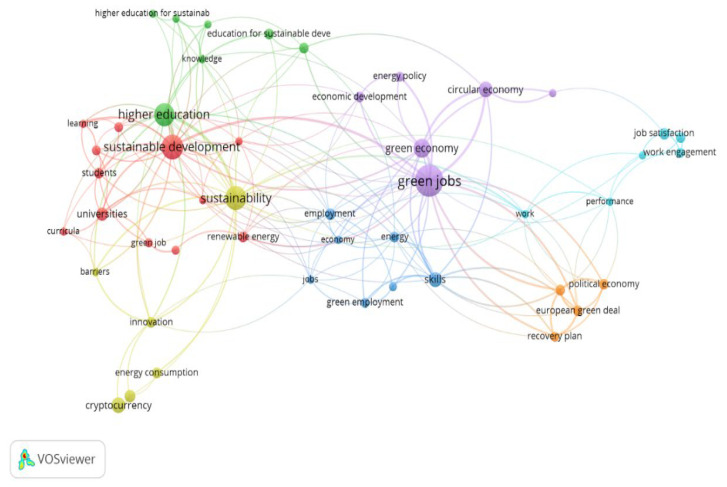
Network of key term associations.

It is noted that there is a significant number of published articles on the topic of “green jobs” during the period under review, but at the same time it can be easily seen that their number has increased significantly in recent years; in a period of 5 years (2018–2022) approximately the same number of articles were published and indexed as in the previous 10 years (2008–2017). In this context, our analysis focuses on the articles published since 2018. Moreover, to ensure that the articles are as relevant as possible and can be easily accessed by the academic community, we have selected those ones indexed both in Web of Science and in Scopus. First, since our analysis focuses on the definition of “green jobs”, we chose papers that use a previous or suggest a new definition for this concept. Second, we looked at the citations for each paper and selected primarily the papers with a high number of citations within Web of Science and Scopus databases. Third, the database with the selected articles was analyzed using the VOSViewer program (version 1.6.18 created by Nees Jan van Eck and Ludo Waltman from Centre for Science and Technology Studies at Leiden University) to identify the correlations between the key terms. The results of the correlation between the key terms are shown in Figure 1.

As showcased in Figure 1, the concept “green jobs” is most often associated with the key term “sustainable development”, with which it has the strongest connection. There are also significant associations between “green jobs” and the following keywords: green economy, circular economy, welfare economy, European Green Deal, energy, renewable energy, economic development, employment. In addition, through sustainable development, the concept “green jobs” relates to other concepts, such as innovation, higher education, education for sustainable development, sustainable development goals, etc. According to these results, we selected the papers in which “green jobs” mainly correlates with topics specific to areas of interest such as green economy, circular economy, labor market, environmental protection, and higher education. Lastly, a selection criterion of a qualitative nature was applied to the articles meeting the above criteria, aiming at the degree to which the papers are relevant to the concept “green jobs”. As a result of applying these criteria, 25 articles summarized in Table 2 were selected.

All the above articles are going to be analyzed in the following sections in order to discover how their authors interpreted and defined “green job” and also to notice with what the topic of green jobs has frequently been associated with.

## 3. ”Green Jobs” Meanings in the Literature

Usually, social scientists as well as economists do not easily agree to a singular definition of the concepts they apply in their studies. Consistent with this empirical assumption, one may say that there is currently no unanimously accepted definition of green jobs among scholars or policymakers [40]. As may be noted, a first association, that almost emerged as a necessity, has been made between the “green jobs” and “decent work” concepts. This connection is the outcome of the hypothesis that green and decent work may be assumed as any provision of services performed under decent conditions following sustainability as main driver.

Since different jobs have diverse impacts on the environment, and decarbonization is a process that takes place gradually, the concept “green jobs” is also under a permanent construction, with no bounded content and meaning [32].

In the literature the “green jobs” term has started to grow more in content in recent years, but the approach to areas that offer such opportunities and specific skills differ among published papers [41] or from one country to another [28]. Meanwhile, the lack of a widely accepted definition comes with several issues [28], including the precise highlighting of areas and the accurate number of such jobs. Valero et al. have shown that a top-down approach that uses a narrow definition of green jobs, considering only those industries or activities that are directly relevant to decarbonization, leads to an estimate of green jobs below 5% of employment in the United States or European economies. The same study emphasizes that when using a “bottom-up” definition, which contemplates jobs directly or indirectly related to the decarbonization, significantly higher percentages can be obtained [41].

Another approach implicates classifying green jobs either as outcomes or as processes [42]. The first perspective goes towards generating environmentally friendly goods or services through these jobs, such as green buildings, clean transport, or solar water heating systems. The second angle assumes that green jobs contribute to greener processes, e.g., by reducing water consumption, controlling air pollution, or improving recycling services. In both cases, there is no question of results based on 100% environmentally friendly production processes or 100% environmentally friendly final goods or services. However, the key element is meeting the criteria of decent work [42].

The prominence of green jobs has been noticed particularly following the emergence of the “Green Jobs Initiative” [2], as well as the economic crisis of 2008–2009 [28].

Following the scrutiny of the selected literature, one can easily presume that many studies have chosen to begun to approach green jobs in line with the “Green Jobs Initiative” description issued by the partnership between the United Nations Environment Program (UNEP), the International Trade Union Confederation (ITUC), the International Organization of Employers (IOE) and the International Labor Organization (ILO). Thus, the studies conducted by: [18,19,21,22,25,26,27,28,30,31,32,33,34] assess green jobs in terms of their ability to decrease the impact on the environment to a level that falls within the range of sustainability. Such jobs involve reducing the consumption of energy, raw materials, and water by adopting and implementing highly efficient strategies aimed at “decarbonizing the economy and reducing greenhouse gas emissions, to minimize or completely avoid all forms of waste and pollution, to protect and restore ecosystems and biodiversity” [32], in line with the “Green Jobs Initiative” [2].

The majority of the reviewed articles rather address the green jobs concept starting from a definition issued by an international body. For instance, Otieno and Ochieng (2018) designate green jobs as any work performed in sectors such as the production of goods and services, agriculture, administration, research and development and the provision of services, which promotes conservation or restoration of the quality of the environment [35]. Following the same reasoning, Traversi et al., shows that by green jobs one may understand all occupational jobs—from agriculture to administration and services—which contribute significantly to preserving or restoring the quality of the environment in terms of eliminating, reducing or mitigating the impact of pollution. The scale of such jobs is driven by the need to adopt methods of producing goods and services in an environmentally friendly manner to limit global warming and irreversible climate change [39].

Drawing upon earlier studies and initiatives, several reviewed papers go back to the concept prior to 2008. For instance, [34] recall that the OECD (1999) defined jobs as “green” if they produced goods and services for measuring, preventing, limiting, and minimizing damage to the environment, water, air, and soil. This study shows that from the OECD perspective, green jobs play a very important role, as they make substantial contributions to solving problems arising from the need for waste recycling, noise pollution, climate change and the restoration and improvement of ecosystems [34]. Therefore, they emphasize that this older definition is quite comprehensive, including all activities that use cleaner technologies, products, and services, with the ability to reduce environmental risks, methods and technologies that minimize pollution and any management practices that permit an efficient use of natural resources [34].

Besides the definition of the “Green Jobs Initiative” [2,22] underscore that the progress made by several institutions of statistics is also worth considering, as the quantification of green jobs by field raises real issues for economists. Thus, the classification made by the US Department of Labor is highlighted, which differentiates “green” employment into three categories (green, new, and emerging, which have improved ecological skills and increased ecological demand) according to the level and the type of impact made by green economic activities and technologies on labor and employment demand. Using a slightly similar reasoning, Sulich and Zema point out that the definition of green jobs can be expressed as an assessing instrument based on the Classification of Activities in Poland, which has its roots in the classifications proposed by the UN, ISIC, and Eurostat [36]. According to [27], green jobs, defined as a category in the Classification of Activities in Poland, can be a factor of: (1) sustainability of the relationship between economy, society, and environment; (2) assessment of the needs of society to be met in terms of environmental protection; (3) limitations of human activity in natural environments; and (4) pro ecological management. The authors point out that the category of green jobs can include those that support the preservation or rehabilitation of the environment in traditional sectors such as production and construction, or in new, emerging sectors such as renewable energy and energy efficiency [27].

It is noteworthy that [25] reveal the progress that has been made by the European Commission (2018) towards a broader definition, according to which a green job “deals directly with information, technologies or materials that preserve or restore the quality of the environment. This requires specialized skills, knowledge, training, or experience (for example, verifying compliance with environmental legislation, monitoring and streamlining the use of resources within the company, promoting, and selling green products and services)” [6]. In this context, green technological innovations seem to be essential for the creation of green jobs [25].

The reviewed articles show that there is no uniformity in the definition of the concept, although some authors start from the same premises outlined in the joint report UNEP/ILO/IOE/ITUC from 2008. For example, in some papers [24], green jobs can mean newly created jobs in the renewable energy sector or in some others studies [20], there is identified the possibility of using a simplistic definition indicating the jobs in the environmental goods and services sector. Bassi and Guidolin have shown that the latter approach does not consider all those employees who use environmentally friendly processes and practices as it assumes that green jobs are synonymous with jobs specific to the circular economy, but that these employees may be defined in many ways [20]. Citing a report of the European Commission from 2015, the authors point out that green jobs include “all jobs that depend on the environment or are created, replaced or redefined in the process of transition to a greener economy” [20].

## 4. Related Research Areas

The topic of green jobs has frequently been associated with the study of sustainable development, the green economy, the circular economy and international agreements on the environment, bringing to the fore the issue of promoting those production and distribution processes that involve the use of procedures, techniques and technologies conducive to the preservation of the qualities of the environment.

Thus, several studies have shown that achieving sustainable development requires rethinking and reformulating the current economic model, which should help promote a transition to a socially and environmentally equitable economy in a context in which the two main challenges facing the 21st Century seem to be the protection of the environment and the transformation of decent work into reality [32]. Following the same line of thinking, ref. [19] points out that the prospects for future growth are important, as the degree of awareness of ecological and environmental issues is constantly growing. Furthermore, the above-mentioned study provides an overview of the economy and economic policies in the framework of the European Environment Agreement and the Spanish Recovery Plan, paying a special attention to the tourism sector and the real opportunities for creating green jobs in the Spanish tourism market and concluding that green jobs are still limited in the Spanish tourism industry.

Moreover, what we discovered reviewing the literature on the development of green jobs is the soft connection between them and higher education institutions. Lee and van der Heijden reveal that there are solid foundations from which we can start by designating universities as key players in the knowledge economy, promoting the green economy through research and development, partnerships, and education. By collaborating with corporations and governments, universities have the opportunity to indirectly influence the demand for green jobs [30]. Green jobs entail a set of green skills that universities grant. At the same time, universities can directly contribute to the creation of green jobs by producing knowledge on the best actions to be taken to combat climate change. Lee and van der Heijden give the example of Cornell University, which cut its carbon dioxide emissions by 30% from 2008 to 2019, implementing a climate action plan and creating green jobs. Two commendable initiatives fall into the same category—Climate Leadership Network and “We Are Still In” [30]. The empirical results of the same study indicate that areas with higher GDP have a higher potential to provide more green jobs; therefore, most policies should be geared towards lower GDP metropolitan areas.

Further studies addressing the issue of green jobs focus on topics closely related to the environment, such as waste management [27], focusing on reducing, preventing, and recycling waste, and producing energy from waste, where the most efficient results can be achieved by implementing waste selective collection. The results of these studies clearly show that the most effective alternative for applying the circular economy model is the specific measures applied by green enterprises in the field of selective collection and recycling of waste, followed by green jobs and, finally, ecological activities assumed by the wide public [27].

Going further, Bassi and Guidolin have investigated small and medium-sized enterprises in the European Union, and have revealed a significant association between green jobs, environmental skills, and embracing circular economic practices [20]. The results show heterogeneity within and between European countries in terms of the employment of circular economic actions by SMEs and confirm that the amount of green jobs and the prevalence of workers with environmental skills play a significant role in determining favorable behavior to the circular economy. In addition, the lack of green jobs has a negative effect on the likelihood of embracing circular economic practices, while the perception of the need for additional environmental skills has a positive effect on the intention to perform actions in the future [20].

In addition, the issue of green jobs has also raised the need for adequate training of people able to perform specific activities. For example, Unay-Gailhard and Bojnec point out the existence of significant differences between green and non-green jobs in terms of skills and human capital [33]. Green jobs require higher levels of non-routine cognitive skills, and a greater dependence on formal education, work experience and training [23]. However, the occupational risk that accompanies green jobs is not to be neglected, the exposure to bioaerosols, endotoxins and particles being particularly relevant [39].

Additional studies have concentrated on an analysis of the factors and conditions that influence the creation of green jobs. Investigating the environmental goods and services sector in 28 EU countries in the years 2009–2019, Sulich and Sołoducho-Pelc pinpoint three of the most important variables for green job creation: (1) private investment, jobs and gross value added related to the sectors of the circular economy (this is the variable with the most significant impact); (2) patents related to recycling and secondary raw materials; and (3) recycling of bio-waste [18]. Ref. [34] show that bureaucracy and lack of investment in infrastructure are real obstacles to green job creation and local green businesses.

In addition, the issue of finding the first job for young people that falls into the category of “green jobs” has been approached from the perspective of comparing opportunities in various economies. Thus, the data compared by [28] for selected groups in the European Classification of Economic Activities (NACE) shows that in both Poland and Belgium, about 15% of young people find their first job in the green jobs sector, but in the Czech Republic, the proportion is much lower (1.83%). It is concluded that a growing emphasis on building the green economy offers excellent employment opportunities for young people looking for their first job.

Song et al., analyzing the supply and demand of green jobs based on data provided by online recruitment services (Ecojob site) on the South Korean labor market from 2009 to 2020, have revealed that green jobs are concentrated in the Seoul and Gyeounggi-do metropolitan areas, where the number of jobs related to water and air quality is high [26]. Their conclusions suggest that green job creation policy should reflect data on regional and sectoral timing, demand, and supply. Creating and matching green jobs is intended to reduce environmental damage, improve environmental quality, and reduce unemployment.

As shown, studies on green jobs have covered different regions of the globe from a geographical perspective. For instance, Unay-Gailhard and Bojnec, combining “top-down” and “bottom-up” analyses of Slovenia’s green economic experience, observed the potential of green economic measures to create green jobs in the agricultural sector, particularly for young people. The study concludes that, at large, for farms that implement green measures, the growth rate of green jobs appears to be much lower than the rate of increase in the capacity to adjust labor force to organic farming activities. In the case of small and medium-sized farms from Slovenia that have adopted agri-environmental measures during 2007–2015, the ability to adjust the workforce to activities compliant to environmental policy does not lead to any significant job creation while the amount of labor used on very large farms and on farms that supply milk increased [33].

The variety of topics related to “green jobs” and the diverse geographical areas covered is furthermore revealed by Martínez-Cruz and Núnez. Their study starts from the uncertainty about Mexico’s ability to pursue an imminent, strong and serious transition in the energy sector. In a discrete choice experiment, a sample of urban residents paying household electricity bills in Aguascalientes, Mexico was studied, with some willingness to pay for both renewable energy sources, and new green jobs in the renewable energy sector and greater availability of solar energy compared to biomass energy [24].

## 5. Conclusions

Recently, the issue of the impact of climate change and the actions needed to combat its negative effects have become a topic of great interest to both researchers and policy makers. One of the tangible results of the academic, social, and political debates is the emergence of the Green Pact, a strategy aimed at sustainable growth, decoupled from the extensive use of resources, and leading to an increase in the quality of life of the individual while reducing the negative impact on the environment. The effective enactment of this strategy requires, among other things, a focus on the green jobs’ initiative, which aims to create decent jobs that contribute to maintaining and restoring the quality of the environment. Beginning from 2008 in particular, the topic of green jobs has attracted the attention of researchers, which has led to a diverse and relatively large number of studies. Following the assumption that, as a consequence, this can raise issues in the systematic identification of contributions to increasing knowledge, the main objective of this research was to analyze the scientific literature to identify definitions and approaches to the concept of “green jobs”, the terms associated with it, the areas of research interest in which the concept is frequently encountered, as well as the main theoretical and practical results obtained in the last five years in research on green jobs.

In the literature, there is currently no universally accepted definition of “green job” [40], and the concept of “green job” seems to be in a state of permanent construction [32]. On the other hand, it seems that most authors choose to address the issue of green jobs starting from the definition given by an international body, such as the definition provided by the partnership between the Program United Nations Environment Program (UNEP), the International Trade Union Confederation (ITUC), the International Organization of Employers (IOE) and the International Labor Organization (ILO). Although progress has been made in the broader definition of green jobs, the lack of a common definition may raise issues as to the exact highlighting of areas and the exact number of such jobs. In addition, the review of the literature reveals that the need to define the concept of “green jobs” is also linked to the desire to highlight the importance of green jobs and the issue of quantifying green jobs by field. The results of the review show that although there is no uniformity in the definition of the concept, there is, however, a convergence towards an initial acceptance of the 2008 UNEP/ILO/IOE/ITUC Report.

Using VOSViewer scientific software our analysis revealed that the term “green jobs” is most often associated with topics such as sustainable development, green economy, circular economy, welfare economy, European Green Pact, energy, renewable energy, economic development, and employment. In any case, the strongest connection is between “green jobs” and “sustainable development”, which highlights a common field and research interest. In addition, through sustainable development, the concept of “green jobs” relates to other concepts that have been hotly debated recently, such as: innovation, higher education, education for sustainable development or sustainable development goals.

It is also worth noting that the main results obtained in studies dedicated to green jobs were aimed at creating green jobs, work–life balance, analyzing the correlations between green business and green jobs or the role of local government in support of green jobs.

The current study provides relevant and promising results, but, on the other hand, focusing only on articles published in the last five years indexed in the Web of Science—Core Collection and Scopus databases—may be considered a limitation of the study since it excludes other academic databases. In this context, the study can be further extended by including papers published in other databases that may also cover the period before 2018.

## Figures and Tables

**Table 1:** Number of articles published on “green jobs”.

Period	2008–2022	2008–2017	2018–2022
Web of Science—Core Collection	245	134	111
Scopus	344	218	126

Source: authors’ processing by using the data provided by scopus.com and webofknowledge.com (accessed on 2 February 2022).

**Table 2:** Reviewed articles.

No.	Article Name	Area of Interest	Terms Associated with “Green Jobs”	Purpose
1	A. Darmandieu, C. Garcés-Ayerbe, A. Renucci, P. Rivera-Torres, “How does it pay to be circular in production processes? Eco-innovativeness and green jobs as moderators of a cost-efficiency advantage in European small and medium enterprises”, *Business Strategy and the Environment*, 31, pp. 1184–1203, 2022 [16]	Circular economy Green economy	Eco-initiatives, Green jobs, Profitability advantage	Providing information on the profile of European SMEs regarding the existence of those processes that are specific to the circular economy
2	C. Liu, Y. Yao, H. Zhu, “Hybrid Salp Swarm Algorithm for Solving the Green Scheduling Problem in a Double-Flexible Job Shop”. *Appl. Sci.*, 12, 205, 2022 [17]	Green energy	Flexible double scheduling Multi-objective optimization Hybrid algorithm Green shop scheduling	Identifying a solution for planning green work schedules to optimize working time and total labor cost relative to the total green index
3	A. Sulich, L. Sołoducho-Pelc, “The circular economy and the Green Jobs creation”, *Environmental Science and Pollution Research* 29, pp. 14231–14247, 2022 [18]	Circular economy	Sustainable development Green goods and services Sustainable development goals	Investigating the environmental goods and services sector in 28 European Union countries in 2009–2019
4	G.E. Arnedo, J.A. Valero-Matas, A. Sánchez-Bayón, “Spanish Tourist Sector Sustainability: Recovery Plan, Green Jobs and Wellbeing Opportunity”, *Sustainability*, 13, 11447, 2021 [19]	Labor market, Employment policies	Political economy Economic policies European Green Deal Recovery plan Welfare economy Spanish tourism and hospitality sector	Analysis of the capacity of the European Environment Agreement and the Recovery Plan to provide real opportunities for green jobs in the Spanish tourism market.
5	F. Bassi, M. Guidolin, “Resource Efficiency and Circular Economy in European SMEs: Investigating the Role of Green Jobs and Skills”, *Sustainability* 13, 12136, 2021 [20]	SME Circular Economy	Resource Efficiency Green Skills Green Economy Multilevel Modeling Circular Economy	Exploring the size and employment potential of the circular economy in SMEs active in the Member States of the European Union Analysis of the link between green jobs and green skills with circular economy practices in European SMEs.
6	F. Dell’Anna, “Green jobs and energy efficiency as strategies for economic growth and the reduction of environmental impacts”, *Energy Policy* 149, 112031, 2021 [21]	Energy sector Environmental protection	Cost–benefit analysis Economic assessment Renewable energy sources Energy policy Impact on labor Markets Adjusted earnings approach Green economy	Investigating the extent to which investments in renewable energy sources can mobilize a country’s economy by exploiting its internal resources both economically, as well as energetically
7	M.V. García Vaquero, A. Sánchez-Bayón, J. Lominchar, “European Green Deal and Recovery Plan: Green Jobs, Skills and Wellbeing Economics in Spain”, *Energies*, 14, 4145, 2021 [22]	Skills and competencies in the labor market Welfare economics	Labor policies European Green Deal Recovery plan Skills Welfare economy	Analysis of the opportunity for green jobs for Europe, with a specific analysis applied to the case of Spain
8	A. Iddagoda, E. Hysa, H. Bulinska-Stangrecki, O. Manta, “Green Work-Life Balance and Greenwashing the Construct of Work-Life Balance: Myth and Reality”, *Energies*, 14, 4556, 2021 [23]	Equilibrium between work and life	Work–life balance Green work–life balance Employee involvement Employee performance in the workplace	Identifying and examining the logical reasons governing “balanced green working life”
9	A.L. Martínez-Cruz, H.M. Núnez, “Tension in Mexico’s energy transition: Are urban residential consumers in Aguascalientes willing to pay for renewable energy and green jobs?”, *Energy Policy*, 110, 112145, 2021 [24]	Renewable energy	Renewable energy sources Fair Energy Transition Discrete Choice Experiment in Aguascalientes Mexico	Analysis of percentage of renewable energy sources in electricity consumption in Aguascalientes, Mexico
10	L. Moreno-Mondejar, A. Triguero, M.C. Cuerva, “Exploring the association between circular economy strategies and green jobs in European companies”, *Journal of Environmental Management* 297, 113437, 2021 [25]	Circular economy	Circular economy The 4R approach Radical innovations vs. incremental innovations Technological and organizational capabilities	Analysis of the relationship between different types of circular economy strategies and the Creation of green jobs by European companies, depending on the degree of novelty and innovation
11	K. Song, H. Kim, J. Cha, T. Lee, “Matching and Mismatching of Green Jobs: A Big Data Analysis of Job Recruiting and Searching”, *Sustainability*, 13, 4074, 2021 [26]	Labor Market Online Recruitment	New Green Deal Job Market Big Data Analysis	Exploring Green Job Supply and Demand in South Korea’s online labor market recruitment services from 2009 to 2020.
12	R. Vesere, S.N. Kalnins, D. Blumberga, “Role of Green Jobs in the Reduction of Waste and Waste Management”, *Environmental and Climate Technologies* 25 (1), pp. 1128–1141, 2021 [27]	Recycling and waste management Environmental protection	Green enterprises TOPSIS Waste management Waste Reduction	Examining the role and place of green enterprises, green jobs, and green social activities in waste management.
13	A. Sulich, M. Rutkowska, Ł. Popławski, “Green jobs, definitional issues, and the employment of young people: An analysis of three European Union countries”, *Journal of Environmental Management,* 262, 110314, 2020 [28]	Green economy	Ecodevelopment European public goods Protected areas	Analysis of the degree to which young people find their first job in the green economy
14	A. Ismail, Z. Kasman, S. Sumarwati, F.A.N. Yunus, N. A. Samad, “The Development of Job Competency For Skilled Technical Worker Towards Green Technology”, *International Journal of Geomate*, 17(59), pp. 216–221, 2019 [29]	Skills and competencies in the labor market	Competence in the Workplace Technically skilled employees Green skills	Analysis of Skills Training for skilled Workers
15	T. Lee, J. van der Heijden, “Does the knowledge economy advance the green economy? An evaluation of green jobs in the 100 largest metropolitan regions in the United States”, *Energy & Environment*, 30(1), pp. 141–155, 2019 [30]	The impact of institutions of higher education on the development of green jobs Connection between knowledge economy and green economy	Green jobs Green economy Knowledge economy Climate action Institutions of higher education	Empirical assessment of the impact of the knowledge economy on the presence of green jobs in the top 100 metropolitan areas in the United States
16	F.-A. Luca, G. Epuran, C.I. Ciobanu, A.V. Horodnic, “Green Jobs Creation—Main Element in the Implementation of Bioeconomic Mechanisms”, *Amfiteatru Economic*, 21(50), pp. 60–74, 2019 [31]	Bioeconomics Environmental protectionSecurity	Sustainable economic growth Bioeconomics Sustainability Environmental behavior	Clarifying divergent views in the literature on the impact of creating green jobs
17	L. J. Rodríguez, “The promotion of both decent and green jobs through cooperatives”, *Boletín de la Asociación Internacional de Derecho Cooperativo*, 54, pp. 115–129, 2019 [32]	Cooperative entrepreneurship	Decent work Cooperatives Sustainable development	Highlighting how cooperatives can be a useful channel for promoting green jobs
18	I. Unay-Gailhard, S. Bojnec, “The impact of green economy measures on rural employment: Green jobs in farms”, *Journal of Cleaner Production* 208, pp. 541–551, 2019 [33]	Agricultural market Environment	Green economy Circular economy Eco-agricultural measures Ranches Network of agricultural accounting data	Analysis of the potential of the green economy to create green jobs in the agricultural sector to identify the identification of measures needed to create green jobs in the agricultural sector for young people in rural areas
19	J.Y. Yong, M.-Y. Yusliza, O.O. Fawehinmi, “Green human resource management. A systematic literature review from 2007 to 2019”, *Benchmarking: An International Journal*, 27(7), pp. 2005–2027, 2019 [13]	Green management of human resources	Green human resources management Green training Management of green human resources	Highlighting relevant information from the literature with emphasis on conceptualization, implementation, and results of green human resources management
20	M. Battaglia, E. Cerrini, N. Annesi, “Can environmental agreements represent an opportunity for green jobs? Evidence from two Italian experiences”, *Journal of Cleaner Production* 175, pp. 257–266, 2018 [34]	Green economy Labor market	Green Environmental agreements Sustainable development Environmental rehabilitation Stakeholder cooperation	Identifying the correlation between green jobs and green business models with industrial conversion in two Italian industrial zones
21	B. Otieno, A. Ochieng, “Green economy in the wastewater treatment sector: Jobs, awareness, barriers, and opportunities in selected local governments in South Africa”, *Journal of Energy in Southern Africa*, 29(1), pp. 50–58, 2018 [35]	Skills and competencies in the labor market	Local administration South Africa	Analysis of the role of local governments in adopting green economy-specific strategies in wastewater treatment in the northern provinces of South Africa
22	A. Sulich, T. Zema, “Green jobs, a new measure of public management and sustainable development”, *European Journal of Environmental Sciences*, 8 (1), pp. 69–75, 2018 [36]	Public management Sustainable development	Sustainable and durable development Efficiency Green jobs Green employment	Developing a tool for effectively measuring green job creation
23	A.O. Afolabi, R.A. Ojelabi, P.F. Tunji-Olayeni, O.I. Fagbenle, T.O. Mosaku, “Survey datasets on women participation in green jobs in the construction industry”, *Data in Brief*, 17, pp. 856–862, 2018 [37]	Analysis of the level of participation of women compared to men in green jobs in the construction industry	N.A.	Questionnaire-based analysis of barriers and socio-economic benefits that can guide policies and actions on attracting, retaining and exploring women’s skills in green jobs in construction in Lagos, Nigeria.
24	D.J., Hess, Q.D. Mai, R. Skaggs, M. Sudibjo, “Local matters: Political opportunities, spatial scale, and support for green jobs policies”, *Environmental Innovation and Societal Transitions*, 26, pp. 158–170, 2018 [38]	Energy-transition policies Politics of transitions in U.S.	Green jobs Economic development Transitions Energy policy Clean technology	Evaluation of media reports on the attitude towards the energy transition policy during the Obama administration
25	D. Traversi, I. Gorrasia, C. Pignata, R. Degan, E. Anedda, G. Carletto, G. Vercellino, S. Fornasero, A. Bertino, F. Filippi, M. Gullo, G. Gilli, “Aerosol exposure and risk assessment for green jobs involved in biomethanization”, *Environment International* 114, pp. 202–211, 2018 [39]	Anaerobic digestion plants Environmental sub-fractionated PM10 and personal PM4.5 Pollution Occupational risk evaluation	Anaerobic digestion Bioaerosol Fractioned PM Endotoxin Occupational exposure	Assessing aerosol exposure for workers in green jobs, focusing on the risk of bioaerosols

Source: author’s work.

## Data Availability

Not applicable.
